# Senescence-Induced Lipidome Alterations in Mesenchymal Stromal Cells

**DOI:** 10.1021/acs.jproteome.5c00355

**Published:** 2025-11-17

**Authors:** Molly E. Ogle, Joseph L. Corstvet, Reesha K. Vayalakkara, Facundo M. Fernández, Johnna S. Temenoff

**Affiliations:** 1 Wallace H. Coulter Department of Biomedical Engineering, Georgia Institute of Technology and Emory University, 313 Ferst Drive, Atlanta, Georgia 30332, United States; 2 School of Chemistry and Biochemistry, 1372 Georgia Institute of Technology, Atlanta, Georgia 30332, United States; 3 Parker H. Petit Institute for Bioengineering and Bioscience, 1372 Georgia Institute of Technology, Atlanta, Georgia 30332, United States

**Keywords:** lipidomics, senescence, mass spectrometry, mesenchymal stromal cells, bioinformatics

## Abstract

Mesenchymal stromal cells (MSCs) show great promise as a clinical treatment for a variety of diseases, but their susceptibility to senescence during culture reduces the therapeutic potential and limits cell expansion. In this study, we explored how MSC lipid metabolism is altered in culture over time using ultrahigh-performance liquid chromatography mass spectrometry. The proportion of cells with senescence-associated β-galactosidase (SA-β-gal) activity was evaluated during 12 days of culture expansion of MSCs from two human donors. Lipid profiles were evaluated in parallel using exact mass and tandem mass spectrometry spectral database matching to generate 237 unique lipid annotations. Lipid abundance generally increased across most lipid classes over serial culture; however, many changes were heterogeneous between donors. Despite donor differences, 12 lipids, including 4 triglycerides (TG), provided discrimination between cultures with less than 10% SA-β-gal+, those with 10–20% SA-β-gal+, and greater than 20% SA-β-gal+ senescence proportion regardless of donor. More specifically, TG composed of long-chain, highly unsaturated fatty acids was strongly associated with higher MSC senescence. These changes in bulk lipid profiles may inform future strategies to monitor early culture senescence during the expansion of MSCs.

## Graphical abstract

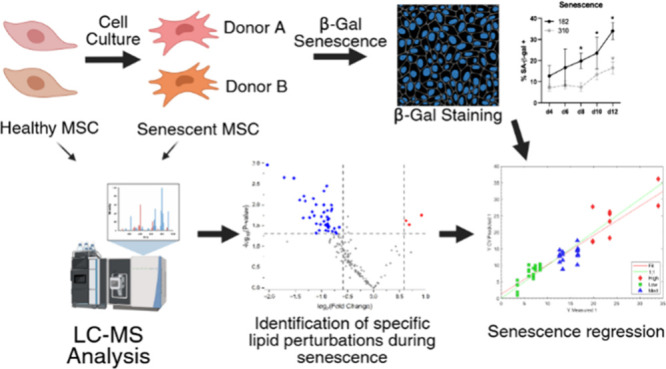

## Introduction

Mesenchymal stromal cell (MSC) therapies have shown preclinical promise for reducing inflammation and improving regenerative processes in a variety of conditions,^1–3^ culminating in their recent FDA approval for treating pediatric graft-vs-host disease.^4^ Both autologous and allogeneic MSC therapies have a low risk of immune rejection and immunomodulatory properties that make them an attractive therapeutic cell source.^5^ MSCs can be harvested from bone marrow, adipose tissue, or umbilical cord blood; however, only a finite number of these primary MSCs can be harvested from one donor. Therefore, in vitro expansion has been employed to achieve large-scale production before clinical application.^3^ The in vitro proliferative capacity of primary cells such as MSCs decreases over extended culture until senescent cell cycle growth arrest.^6,7^ Limited expansion in culture due to senescence, as well as batch-to-batch variability during scale-up, is a major challenge in MSC clinical development^8,9^ and biomanufacturing.

Senescence is characterized by increased expression of cell cycle-related genes p16^INK4A^ (*CDKN2A*) and p21 (*CDKN1A*),^10^ activation of lysosomal senescence-associated β-galactosidase (SA-β-gal), cell morphological changes, and increased expression of inflammatory senescence-associated secretory factors.^11,12^ Although senescent MSCs can remain viable and metabolically active in culture, they may not maintain their therapeutic properties.^13^ Senescent MSCs can have impaired pro-regenerative activity, altered paracrine secretions, and reduced differentiation and migration potential.^13^ To enable the optimization of MSC biomanufacturing methods, early measures of senescence (at the bulk/batch level) could provide crucial information for production and quality control of MSCs expanded in large-scale culture.

Cellular metabolism is a highly sensitive process that is altered in senescence.^14^ Metabolomic analysis by mass spectrometry allows the evaluation of subcellular-level features of a cell population that may be useful in understanding the biological state of the cell culture. Metabolic changes within the cell frequently precede measurable RNA- and protein-level changes and therefore could provide a potential early assessment tool for cell monitoring and quality control.^15^ Metabolic fingerprints of MSCs have been associated with therapeutic behaviors.^16^ Therefore, metabolic markers may support early prediction of the fitness of a heterogeneous cell population. Lipids are an important class of metabolites as they are important regulators of cell homeostasis, health, cell polarity, asymmetric division, intracellular signaling, and energy generation.^17^

MSCs can undergo senescence in the body during the natural process of aging. MSCs isolated from aged rat bone marrow (15–18 months) had metabolite and gene changes associated with lipid metabolism relative to MSCs from young bone marrow (1–2 months); however, this study did not evaluate the relationship with senescence. MSCs from the older group had several differentially represented metabolites associated with glycerophospholipid metabolism, linoleic acid metabolism, and biosynthesis of unsaturated fatty acids.^18^ Studies of lipid metabolites of human MSCs showed that increasing culture passages were associated with alterations in glycerophospholipid and sphingolipid metabolism.^19^ Therefore, MSC lipid metabolism may be altered in aging and over time in culture; however, the relationship with senescence is less clear. In the current study, we evaluated bulk lipid metabolites over a serial culture as potential markers for senescence in MSC cultures.

Here, we sought to explore how human bone marrow MSC lipid metabolites are altered over time in culture and in relation to the proportion of senescent cells in the culture. We hypothesized that the lipid metabolic profile of MSCs in culture is associated with the percent senescence of the culture population. To support our objective, we monitored two biologically distinct human MSC lines over a duration of three serial culture expansions on tissue culture plastic (TCP). We evaluated the lipidomic profile and population senescence of these MSCs by ultrahigh-performance liquid chromatography mass spectrometry (UHPLC-MS) and staining in parallel cultures for SA-β-gal activity. Lipid profiles were constructed using exact mass and tandem mass spectrometry (MS/MS) spectral database matching to generate 237 unique lipid annotations. Lipid abundance relative to the percent SA-β-gal of the culture was used to conduct multivariate analysis to pinpoint lipid features associated with higher percentage senescence in the cultures in order to determine putative lipid markers. Finally, we began to validate these putative markers by culturing a separate batch of each MSC line, following identical sampling and analysis techniques.

## Experimental Procedures

### Human MSC Culture

Two human bone marrow MSC lots (Donor 182, Donor 310, RoosterBio) were culture expanded in RoosterNourish media (RoosterBio) for two passages prior to liquid nitrogen cryopreservation in CS10 freezing medium (Cryostor). Cells were revived from frozen storage and rescue cultured in standard TCP flasks and low-glucose DMEM (Gibco) containing 10% fetal bovine serum (FBS) (Omega Scientific) and 1% antibiotic/antimycotic (Corning) for 4 days prior to seeding into studies. Cells were harvested with TrypLE-Express (Invitrogen) by the manufacturer protocol and were seeded at a density of 10,000 cells/cm^2^ on standard six-well tissue culture plates for three successive passages of 4 days each. Samples were harvested every 2 to 4 days for 12 days total. Parallel cultures were used for the staining of SA-β-gal activity. These methods were used to perform two culture “cohorts” of the same donors. Results from the first cohort were used to build the partial least-squares regression (PLSR) models and identify key discriminatory lipids, while the second culture cohort was used to validate findings with independent culture, sample processing, and analysis.

### Reagents

LC-MS grade methanol, water, isopropyl alcohol (IPA), and ammonium acetate (NH_4_Ac) were purchased from Sigma-Aldrich (Sigma-Aldrich Corporation, St. Louis, Missouri, USA). Ammonium formate, formic acid (99.5+%), acetonitrile (ACN), and ammonium acetate (NH_4_Ac) were purchased from Fisher Chemical (Fisher Scientific International, Inc., Pittsburgh, Pennsylvania). These reagents were used as solvents for extraction, to reconstitute sample extracts, and to prepare chromatographic mobile phases. An isotopically labeled lipid splash mix used as an internal standard (ISTD) was purchased from Avanti Research (Avanti Research, Birmingham, Alabama).

### MSC Harvest and Metabolite Extraction

MSCs for metabolomics analysis were harvested by detachment with TrypLE Express followed by a wash with cold 155 mM ammonium acetate, centrifugation, and resuspension in ammonium acetate at a concentration of 80,000 cells per 50 μL. Aliquots containing 80,000 cells were then quenched with 200 μL of −80 °C HPLC-grade methanol and frozen at −80 °C. A standard isopropanol lipid extraction was performed by centrifuging samples at 16,000 RPM for 5 min to pellet cells, the supernatant was removed, and then 600 μL of isopropanol was added to each sample in addition to 50 μL of 500 μm glass beads. Samples were homogenized using a TissueLyser II (Qiagen, Germantown, Maryland, USA) at 30 Hz for 5 min, samples rotated, and homogenization repeated for an additional 5 min. Following homogenization, samples were centrifuged at 21,000*g* (Labconco) for 5 min, the supernatant was collected in new vials, the beads were washed with 200 μL of fresh IPA and centrifuged again, and the remaining supernatant was collected. The samples were then dried by vacuum centrifuge for approximately 3 h, and extracts were stored at −70 °C until the day of LC-MS analysis. On the day of analysis, each sample was reconstituted in 8 μL of IPA/ISTD and 4 μL of 200 mM ammonium acetate, resulting in 4 μM ISTD, prior to LC-MS runs. Blank samples were prepared by performing the lipid extraction described above using only IPA. A quality control (QC) sample was created by pooling equal aliquots of each sample.

### SA-ß-gal Staining

Colorimetric staining of SA-ß-gal was conducted using the Cellular Senescence Staining Kit (Cell Biolabs, Inc.) by the manufacturer’s protocol. Briefly, at indicated time points, MSCs were washed with phosphate-buffered saline (PBS from Thermo Fisher Scientific, USA) three times and then fixed using the included fixation buffer. Following washing with PBS, cells were incubated with the staining solution at 37 °C overnight. Staining solution was removed, and cells were washed and counterstained with Hoechst nuclear stain (Thermo Fisher Scientific, USA). Cells were covered with 50% glycerol (Fisher Chemical) in PBS and imaged by a light microscope (Nikon Eclipse TE2000-U). Percent senescence was determined by counting cells positive for SA-ß-gal relative to the total cell number. For each group, four replicate wells were imaged and quantified per group.

### LC-MS Experiments and Data Analysis

Samples were analyzed using reverse-phase LC-MS on an Orbitrap ID-X Tribrid mass spectrometer (Thermo Fisher Scientific, Waltham, Massachusetts) coupled to a Vanquish Horizon LC instrument (Thermo Fisher Scientific, Waltham, Massachusetts). Reverse-phase separations were achieved using an Accucore C30 column (150 × 2.1) (Thermo Fisher Scientific, Waltham, Massachusetts) at 50 °C with a flow rate of 0.4 mL/min flow rate. Mobile phase A contained 40% water, 60% ACN, 10 mM ammonium formate, and 0.1% formic acid. Mobile phase B consisted of 90% IPA, 10% ACN, 10 mM ammonium formate, and 0.1% formic acid. Mass spectrometry data was collected in positive ion mode. A 2 μL injection volume was used per injection, and a single injection was performed from each sample. Pooled QC injections were added after every eight sample injections to correct instrumental drift during the analysis. Sample order was randomized to minimize effects of instrumental drift and other unknown confounding variables in the study. Data-dependent acquisition (DDA) was employed to obtain fragmentation spectra (MS^2^) for detected lipids. Full-scan MS data was obtained at 120,000 mass resolution, followed by a collection of MS^2^ of selected precursor ions with an isolation window of 0.4 *m*/*z*. Ions were fragmented using stepped normalized collision energies of 15, 30, and 45 with HCD, and a CID collision energy of 35 was used for MS^2^ spectral collection.

Spectral features from the raw files underwent retention time alignment, peak picking, peak area integration, and QC-based correction using Compound Discoverer v3.3 (Thermo Fisher Scientific). Blank injections were used to remove background features from sample runs. Signals that were lower than twice the intensity of the signal in the blank were removed from sample spectra. Features that were present in >50% of pooled QC samples were retained, while features that did not meet these criteria were filtered out of the data set. This resulted in a list of 2240 features in the first MSC cohort and 2765 features in the second cohort. These lists were further filtered by removing features with corrected QC area %RSD >10% and features that lacked sufficient MS^2^ spectral data. Annotations were determined based on MS^2^ fragmentation patterns by matching against an in-house lipid spectral database. Features that did not match the local database were annotated from exact mass and MS^2^ matching to LipidMaps or the Human Metabolome Database. Final lists of 237 annotated lipids in the first cohort and 40 annotated triglycerides from the second cohort were used for comparative analysis and predictive model creation. Complete lists of annotated lipids are provided in the Supporting Information.

### LC-MS Data Analysis

Partial least-squares discriminant analysis (PLS-DA) and PLSR modeling were performed using the PLS_Toolbox v.9.2 (eigenvector Research Inc., Manson, Washington) for MATLAB (MathWorks, Natick, Massachusetts). Autoscaling of the data was performed prior to analysis, and venetian blind cross-validation with 10 data splits was applied for developing all PLS models. Fold change and statistical significance analysis between sample conditions was performed using Metaboanalyst v6.0 (http://www.metaboanalyst.ca/MetaboAnalyst). To investigate the significance of fatty acid chain length and degree of unsaturation to senescence level, double-bond equivalence versus carbon number plots (DVCs) were created for several lipid classes. Volcano plots and DVCs were visualized using OriginPro 8 (OriginLab, Northampton, Massachusetts). Cluster maps were generated by using the Plotly graphing library for Python.

## Results

### Human MSCs Undergo Senescence in Culture

MSC samples were collected every 2 days over 12 days of culture for evaluation of the lipid metabolic profile and the proportion of cells undergoing senescence (Figure 1A). Primary human MSCs can exhibit batch-to-batch and donor differences in culture proliferation, doubling time, and the rate of development of senescence.^20^ Two human donor cell lines were evaluated in this study. Over time in culture, a larger percentage of cells stained positive for SA-β-gal activity in both donor cell lines (Figure 1B,C). Cells from Donor 182 increased in the proportion of senescent cells in the second and third passages (days 8–12), whereas Donor 310 did not increase in senescence until day 12 (Figure 1C). In agreement with these data, Donor 310 increased in population doubling level from day 4 through day 12. Donor 182 did not have an increase in the population doubling level until days 8 to 12, indicating low proliferation (Figure 1D).

**Figure 1:**
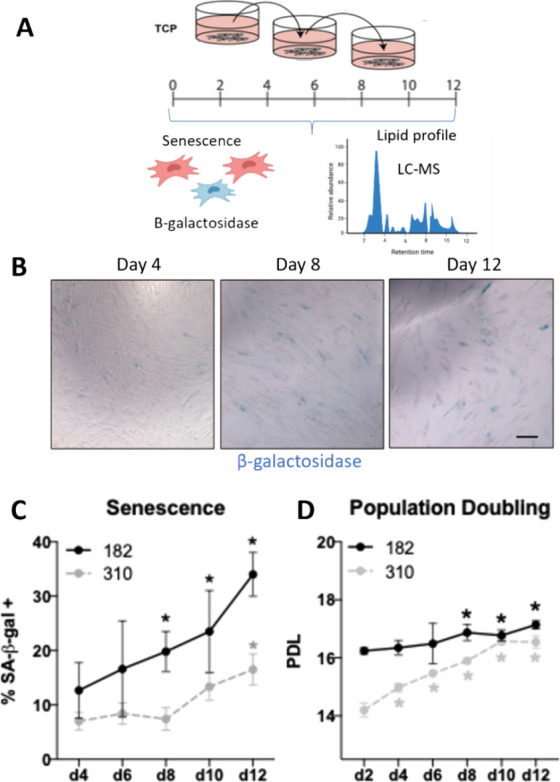
MSC undergoes senescence during standard culture. (A) Schematic of experimental study workflow. TCP, tissue culture plastic. (B) Representative brightfield images of human MSCs stained with senescence-associated β-galactosidase (blue), scale bar 20 μm. (C) Percent β-galactosidase staining by day of culture. (D) Population doubling level for each donor cell line. Data are presented as mean ± SD, *n* = 3–4; one-way ANOVA, *p < 0.05 vs. first time point. Schematics were created in Microsoft PowerPoint or BioRender.

### MSC Lipidome Changes during Senescence

Analysis of MSC cell pellets of equal cell number by reverse-phase UHPLC-MS yielded clear metabolism differences during standard culture (Figure S1A) as well as differences between donors (Figure S1B). From this analysis, a data set of 237 lipid annotations was obtained by exact mass and MS^2^ spectral database matching. Phosphatidylcholines (PC) were the most annotated lipid class with 54 unique annotations, followed by sphingomyelins with 25 annotations and 19 phosphatidylethanolamines (PE) (Figure 2A). This feature list was used to conduct both supervised classification and regression methods to determine which observed lipids were altered most significantly during senescence (Table S1).

**Figure 2:**
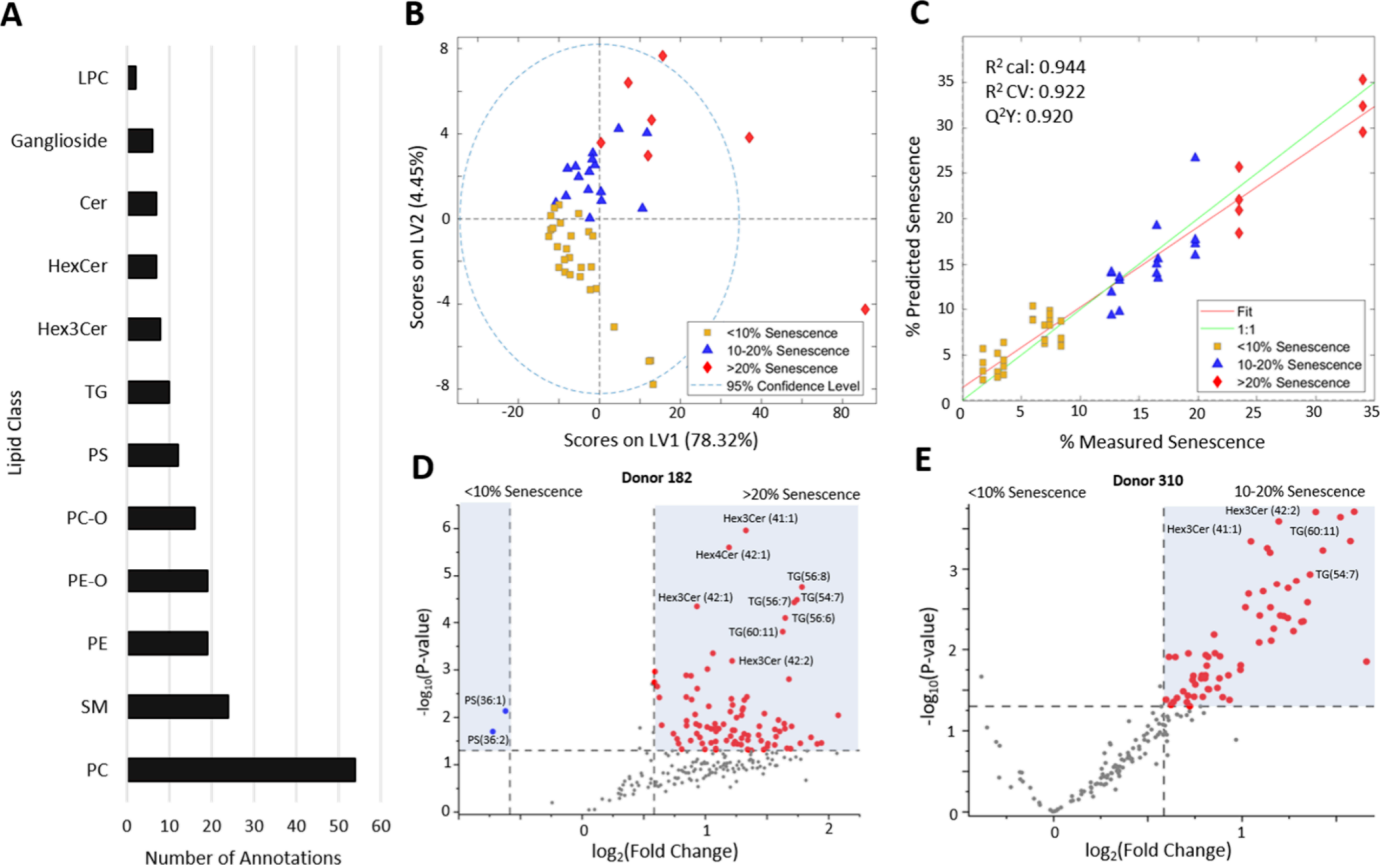
MSC lipid profile alterations at increased senescence levels. (A) Number of MS/MS annotations for each lipid class observed in the study. (B) PLS-DA score plot evaluating the lipidome of two MSC donors over culture time. Individual points coded by % senescence of the culture: <10% (yellow square), 10–20% (blue triangle), >20% (red diamond). *n* = 3–4 replicates per culture and time point, donor cell lines. LV, latent variable. (C) PLSR predicted vs measured senescence based on the lipid profile. (D) Volcano plot identifying key lipids differentiating between lower senescence (<10%) and greater senescence (>10%) in Donor 182 and (E) Donor 310. LPC, lysophosphatidylcholine; Cer, Ceramide; HexCer, hexosylceramide; Hex3Cer, trihexosylceramide; TG, triglyceride; PS, phosphatidylserine; PC-O, ether-linked phosphatidylcholine; PE-O, ether-linked phosphatidylethanolamine; PE, phosphatidylethanolamine; SM, sphingomyelin; PC, phosphatidylcholine; *R* ^2^ cal, calibration; *R* ^2^ CV, cross-validation.

Because each donor underwent differing levels of senescence over the 12-day culture (Figure 1C), MSC samples were classified based on the percent senescence, rather than time in culture. MSC cultures were classified into three groups, those that exhibited <10% positive SA-β-gal staining, between 10 and 20%, and >20% positive staining. PLS-DA was first performed with all 237 annotated features. Cultures from the three levels of senescence exhibited good class separation for each condition, as shown by the PLS-DA score plots, suggesting detectable differences in the corresponding lipidomes (Figure 2B). The three senescence classification conditions were separated along latent variable 1, accounting for 78.32% of variance in the data set. The percentage of MSCs undergoing senescence in each sample was predicted using all of the annotated features by a PLSR model (Figure 2C). True senescence values were determined from the SA-β-gal staining assay, and the predicted senescence value was obtained from the PLSR model. Using all 237 features yielded a strong predictive capability while explaining a significant amount of variance, with *Q* ^2^ *Y* = 0.920, indicating that the assessed MSC lipidome (237 annotated features) was perturbed during senescence. The most discriminatory of these lipids were visualized for each donor cell line using volcano plots between low senescence samples (<10% senescence) and higher senescence samples (>10% senescence) (Figure 2D,E). The abundance of most lipids increased in higher senescence samples compared with lower senescence samples. These lipids represented diverse lipid classes, and the changes were consistent across donors. Triglycerides (TG) and trihexosylceramides (Hex3Cer) had a pronounced increase with senescence compared with other lipid classes.

### Biologically Distinct MSC Donors Exhibit Differing Lipidomic Profiles

We next evaluated the differences in the lipid profile between the two MSC cell lines to determine if differences at the donor level were observed. When combining data from all time points for each donor, we identified heterogeneity between the donors that was not related to the level of senescence. Several PC variants were more abundant in Donor 182, while certain ether-linked phosphatidylcholines (PC-O) were elevated in Donor 310 samples (Figure 3A). In addition, for samples with <10% SA-β-gal+ staining, Donor 182 had significantly increased abundance in most lipids compared to Donor 310. PE was the principal lipid class driving these donor differences (Figure 3B). In 10–20% SA-β-gal+ stained samples, Donor 310 has an increased abundance of multiple PC-O and gangliosides compared to Donor 182 samples (Figure 3C). TGs were not largely different between the donors when evaluated at a similar percentage of culture senescence. Analysis of fatty acid chain lengths and unsaturations also exhibited different lipid abundances during culture (Figure S2A–E). Increased lipid abundances, specifically with long fatty acid chains in Donor 310, was observed, while this trend was inverted in Donor 182. Specific lipids that shift during senescence are shown in Figure S3A and Table S3 for Donor 182, while Figure S3B and Table S4 describe these lipids for Donor 310.

**Figure 3:**
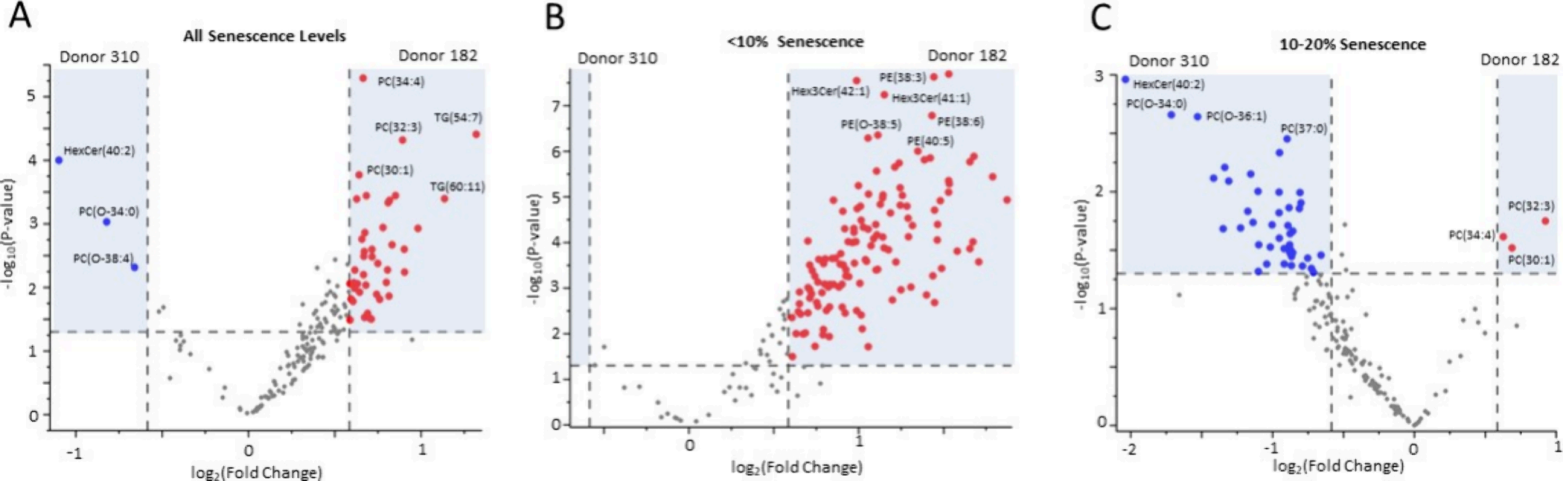
Donors exhibit heterogeneous lipid profiles regardless of the senescence level. Volcano plots identify significant lipidome differences between Donors 182 (right, red) and Donor 310 (left, blue) for (A) all time points combined, (B) <10% senescence levels, and (C) 10–20% senescence levels. Lipid differences were considered significant if they exhibited a fold change >1.5 and *P*-value <0.05. PC, phosphatidylcholine; PE, phosphatidylethanolamine; TG, triglyceride; HexCer, hexosylceramide; Hex3Cer, trihexosylceramide.

### TG Are Associated with Increased Percent Senescence in MSC Cultures

In a heatmap visualization of samples from all donors and time points classified by percent senescence, TG showed an increasing trend. Lipid abundances were normalized to the sample with the highest senescence, which had the highest lipid abundances for every class. An increased abundance of most lipid classes was observed during serial culture; however, TGs were the only class to exhibit a relatively linear abundance increase across passages (Figure 4A). A detailed progression of relative abundances for each lipid class shows this increase of TG across culture days for both Donors (Figure S4A,B) while fold change values show that this progression is rather unique to TG (Figure S4C). Samples with 23.5% positively senescent cells had the highest abundance of TG and Hex3Cer. We investigated the lipids that varied between MSC cultures with <10% senescence and cultures with 10–20% senescence to determine the important features discriminating these conditions regardless of donor (Figure 4B). Twelve lipids were identified to differentiate <10% and 10–20% senescence levels (Table S5). Of these 12, nine were TG, including TG(54:7), TG(56:8), TG(56:6), and TG(56:8), whereas the remaining three discriminating lipids were Hex3Cer(41:1), Hex3Cer(42:0), and Hex3Cer(42:1).

**Figure 4:**
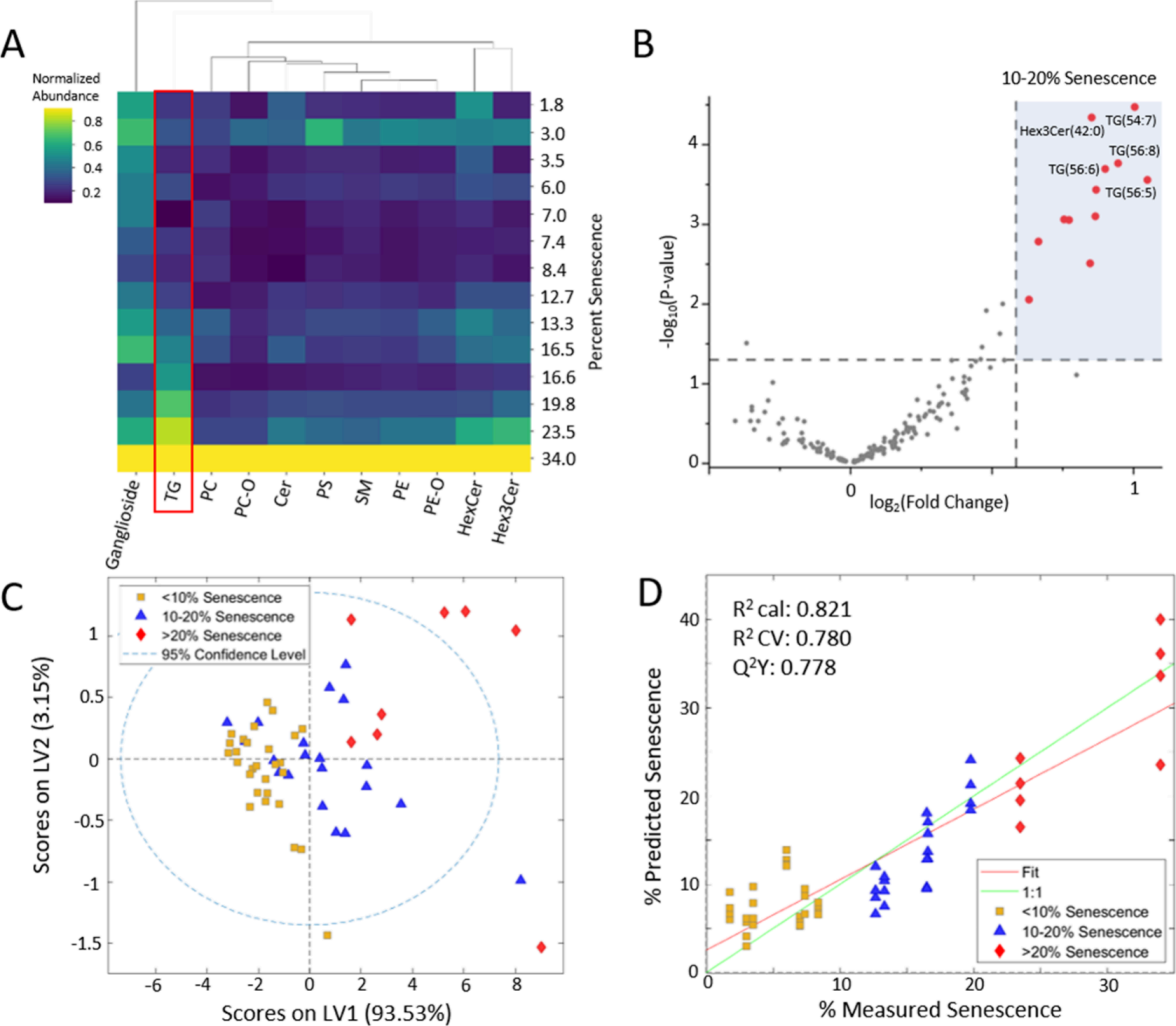
TGs increase as MSC senescence increases. (A) Heatmap displaying normalized abundance of each lipid class at varying senescence percentages. (B) Volcano plot of differential features observed between 10–20% and <10% senescence MSC from both donors. (C) PLS-DA score plot exhibiting clustering of each senescence level and (D) PLSR predictive model created exclusively using the abundances of annotated triglycerides. LV, latent variable. *R* ^2^ cal, calibration; *R* ^2^ CV, cross-validation.

Next, we restricted the analysis to include only annotated TG and trained a PLSR predictive model of the TG profile in both MSC donor lines versus SA-β-gal senescence data. The scores plot of this model showed good separation of <10, 10–20, and >20% senescence along latent variable (LV1), which accounted for 93.33% of the variance in the model (Figure 4C). We used these features exclusively to predict percent senescence of MSC samples, achieving modest predictive capability (*Q* ^2^ *Y* = 0.778) using this model (Figure 4D).

To further validate this finding, an independent culture and lipidomic analysis was conducted with the same MSC donors (second culture cohort of the same donors). To demonstrate the potential efficacy of TG as a senescence predictor, a PLSR model was created to measure the predictive capability of these lipids (Figure 5A) and PLS-DA analysis was performed to identify sample class clustering (Figure 5B). This analysis was performed using all 40 confirmed TG annotations from the second cohort study (Table S2). The PLSR model was able to predict percent senescence with *R* ^2^ = 0.859, describing a large proportion of the variance, further strengthening the hypothesis that TG are indicators of MSC culture population senescence.

**Figure 5:**
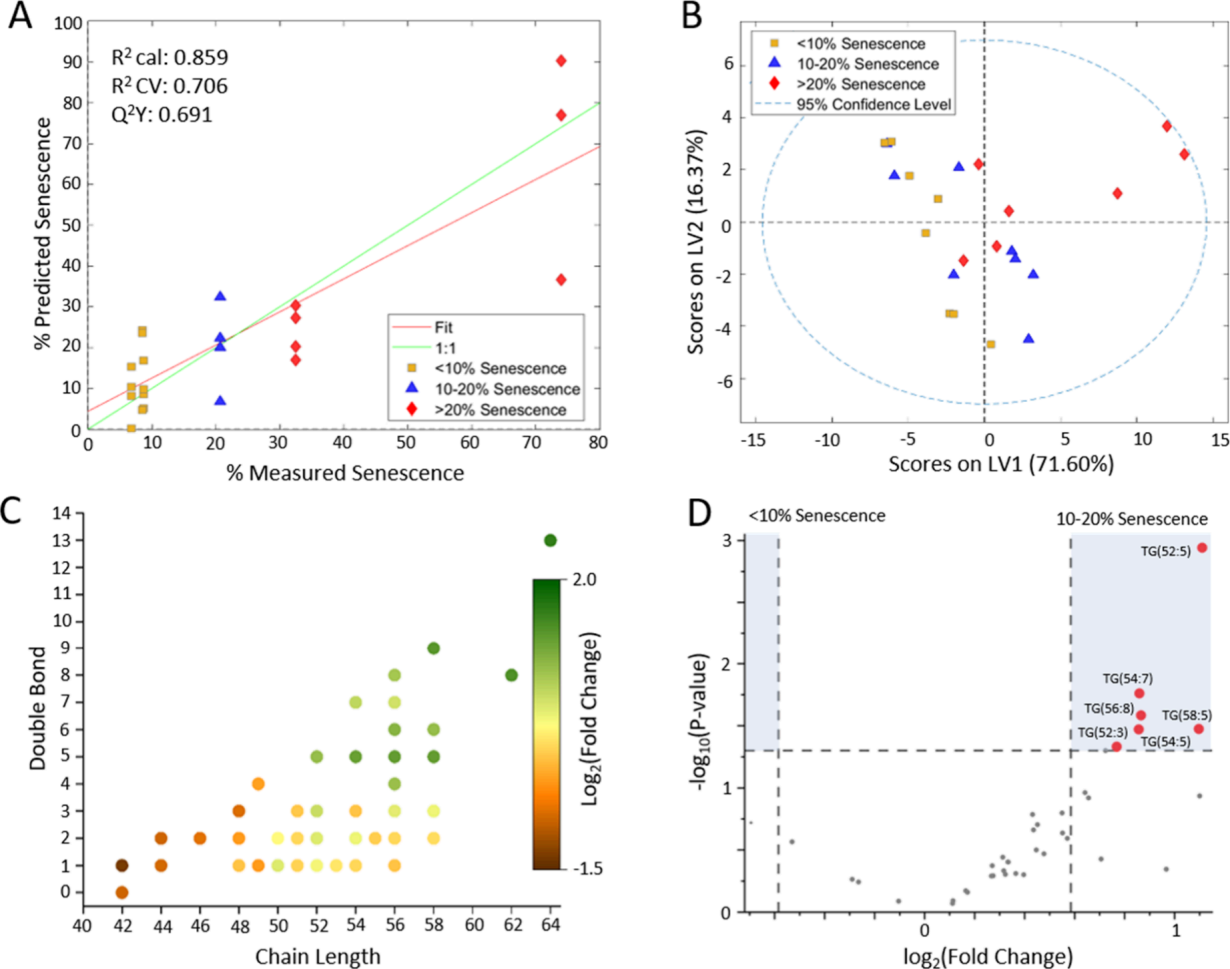
Long-chain TGs are associated with senescence. (A) PLSR model relating percent senescence against TG abundances (3 LV model) and (B) PLS-DA scores plot showing grouping between three senescence levels, using only information regarding TG. (C) DVC showing observed fold change between 10–20% and <10% senescence samples for TG of various chain lengths and degrees of unsaturation. (D) Volcano plot identifying key TG distinguishing 10–20% from <10% senescence MSC.

### TG with Higher Carbon Number and Degree of Unsaturation Are Associated with Increased Senescence

Finally, we evaluated chain length and saturation of the TG we identified. Longer-chain TG, including many with carbon chain length of 52 or greater, were significantly associated with higher senescence levels. In addition, TG with five or more unsaturations were increased in abundance in 10–20% senescence samples compared to <10% senescence (Figure 5C). This trend was similarly visible in a volcano plot, as most features more abundant in 10–20% senescence samples were highly unsaturated and overall TG abundance appeared to skew higher in 10–20% senescence samples (Figure 5D). All these trends agreed with our findings from the first sample cohort.

## Discussion

This work leveraged high-resolution mass spectrometry for characterizing MSC lipid profiles over three passages in two distinct donors to better define lipid metabolites associated with culture-induced senescence. After the first culture was performed, trends and findings were confirmed with a second set of cells from the same donors. From these studies, we found that the overall lipid profile of MSCs could be used to discriminate the senescence level of a culture (rather than simply passage number), regardless of how senescent the cells became over three passages (Figures 1 and 2). The identification of features that are present early in culture that can aid in the prediction of culture senescence is crucial for scale-up manufacturing of these cells. It would allow clinical cell production teams to preserve resources by identifying failed batches early, or potentially intervene to reduce senescence and produce a more potent product.^21^ Such markers could be used to both monitor culture batches and release criteria and screen and optimize new biomanufacturing methods.

We observed an increased abundance of most lipids as MSCs remained in culture longer. Cell enlargement is a morphological change characteristic of MSC senescence;^22^ therefore, the observed increase in lipid abundance across classes may be partially driven by cell enlargement. Larger cells demand more membrane lipids, which may drive the increased abundances of PC and PC-O, as described in other MSC cultures.^19,23^ As the most abundant membrane lipid in eukaryotic cells, PCs act as key modulators of MSC membrane structure and assume roles in cell cycle regulation.^19^ Although we observed global changes in PC and PC-O with culture time, when comparing the two MSC cell lines at the same senescence level, several lipids in the PC and PC-O classes were different between donors, indicating that there may be underlying heterogeneity between different donors. Further work with additional cell lines and genetic backgrounds would help to address this question. In this work, to distinguish the lipids more robustly associated with senescence, we did not focus on metabolites that varied between donors.

In contrast, TG showed similar trends between both donors, suggesting that as a class they may be a more robust marker of cellular fitness and senescence across multiple patients (Figure 4). The TG lipid class, which was highly represented in the most senescent cells, was sufficient to distinguish cultures with less than 10% senescence from those with 10–20% senescence. In particular, several TG and Hex3Cer were perturbed between <10 and 10–20% senescence samples, such as TG(54:7), TG(56:8), and Hex3Cer(42:0). Hex3Cer were found at higher abundances in plasma of human dementia patients compared to healthy control plasma,^24^ a disease that is closely linked to advanced senescence progression in multiple brain cell types.^25^ Our findings also agree with previous research as studies in human fibroblasts have also identified upregulation of both TG and Hex3Cer metabolism during development of senescence.^26^ More specifically, we found that the TGs with the strongest association to senescence were TG composed of long and highly unsaturated fatty acids. A previous senescence lipidomics study in a fibroblast cell line observed an accumulation of TG, mainly composed of polyunsaturated fatty acid chains.^27^ This upregulation of TG storage coincides with an increased lipid droplet accumulation, a morphological change observed across multiple studies in various cell types exposed to cellular stress conditions, both in vivo^28^ and in vitro.^29,30^ The observed perturbations to TG metabolism were validated with a second independent culture and analysis (Figure 5), suggesting that a TG lipid panel could be constructed to assay senescence in MSC cultures.

Triglycerides are among the few lipid classes that have roles not associated with the cellular or mitochondrial membrane.^31^ Our observed association between senescence level and accumulation of highly unsaturated TG may be a defense mechanism within the cell. Unsaturated fatty acids are prone to oxidation so diverting these to lipid droplets and away from membranes could reduce the creation of lipid peroxides, limiting membrane damage under the oxidative stress of senescence.^32^ While we identify TG as a marker of cellular aging, it remains unclear whether these lipids also act as a driver of senescence. Future studies will further evaluate how TG is mechanistically related to senescence in MSC cultures.

Our study is not without limitations. We found that lipid profiles did distinguish MSCs from cultures with differing senescence; however, these observations are based on bulk lipidomic profiles, so our data do not inform specific individual changes in senescent cells, but rather the status of the combined pool of cells where a percentage of those cells are senescent. Despite this limitation, lipid metabolomics was sensitive enough to identify the population percentage of senescence in the cultures in this study. As metabolomics at the single-cell level becomes available, future studies using cell sorting or similar techniques may further distinguish whether the observed changes are specifically enriched in the senescent or nonsenescent cells in the cultures.

Another limitation is that, for this initial screening study, due to the technical complexity of sampling every 2 days across multiple passages, here we evaluated cells from two biologically independent donors over an extended (longitudinally sampled) culture period (six separate time points) with an independently conducted validation study, which is particularly relevant given the batch-to-batch variation often found in expansion of MSCs.^8,9^ Within this design, we focused on the onset of senescence under normal culture conditions and the relation of specific lipidomic features with the accumulation of senescent cells in a culture. Interestingly, across the independently conducted studies, we observed similar changes in TGs that were related to the % senescence of the culture, regardless of the donor or batch. These findings support our conclusions; however, we acknowledge that follow-on studies using more targeted approaches to validate these putative markers across more donors and batches would be needed to definitively relate the lipid species identified here with MSC senescence. A previous larger study of MSC metabolites showed that MSCs exhibit lipidomic heterogeneity overall, but select lipids can be associated with cell behavior, including immunomodulatory potency.^20^ The validation study run here had higher percentages of senescence using the same cell lines and the same conditions, however, the relationship of senescence and TGs was preserved, supporting further investigation of lipidomic profiles in the context of culture-induced senescence.

## Conclusions

In this study, we sought to observe lipidome perturbations as senescence progressed in MSC cultures using high-resolution mass spectrometry using cells from distinct donors. A global increase in all lipids was identified, with TGs specifically becoming more abundant in highly senescent samples, regardless of the donor. Highly unsaturated fatty acid TG appeared as the most characteristic of senescence and was thus used in a regression model to predict the percentage of senescent MSC in culture. This model showed excellent performance at predicting the senescence level of MSC cultures and was comparable to models trained using all annotated lipids. These results provide insight into early changes in lipid metabolism in cells that undergo senescence. In addition, further validation of the model developed in this study may allow for earlier senescence detection in MSC culture and thus optimize culture conditions to improve MSC potency for a wide range of clinical applications.

## Supplementary Material



## Data Availability

Data generated in this study are available through the NIH Common Fund’s National Metabolomics Data Repository (NMDR) Web site, the Metabolomics Workbench https://www.metabolomicsworkbench.org with the project ID PR002407 and study ID ST003849. The data can be accessed directly via its project DOI: 10.21228/M8HC3G.

## Notes

Methods; (Figure S1) MSC undergoing senescence at varying times during standard culture; (Figure S2) abundance alterations observed in specific lipid classes during MSC senescence; (Table S1) all lipid annotations, *m*/*z*, and retention times measured for the first MSC cohort; (Table S2) annotations and *m*/*z* of all TG observed in the second MSC cohort; (Figure S3) volcano plots of Donor 182 and Donor 310 comparing >10 to <10% senescence MSC; (Table S3) key features to distinguish highly senescent samples from <10% senescence samples in Donor 182; (Table S4) key features distinguishing 10–20% senescent samples from <10% senescent samples in Donor 310; (Table S5) significant features in 10–20% senescence from Figure 4B; and (Figure S4) average fold change of each lipid class at every culture day, compared to the abundance of that class in day 0 samples and a table of select values (PDF)
