# Supplementary material for: Senescence-Induced Lipidome Alterations in Mesenchymal Stromal Cells
Source: J Proteome Res. 2025 Nov 17;24(12):5986–94. doi: 10.1021/acs.jproteome.5c00355 (PMC12687314; doi:10.1021/acs.jproteome.5c00355)
Supplement: Supplementary file 1 [file pr5c00355_si_001.pdf]

Supplemental Information for:

Senescence-Induced Lipidome Alterations in Mesenchymal Stromal Cells

Molly E. Ogle[1,3,†], Joseph L. Corstvet[2,†], Reesha K. Vayalakkara[1], Facundo M. Fernández[2, 3], Johnna S. Temenoff[1,3,*]

[1]Wallace H. Coulter Department of Biomedical Engineering, Georgia Institute of Technology and Emory University, 313 Ferst Drive, Atlanta, GA, 30332 USA.

[2]School of Chemistry and Biochemistry, Georgia Institute of Technology, Atlanta, Georgia 30332, USA.

[3]Parker H. Petit Institute for Bioengineering and Bioscience, Georgia Institute of Technology, Atlanta, GA, 30332 USA.

Table of Contents:

S0. Additional methods.

Figure S1. MSC undergo metabolism changes during standard culture.

Figure S2. Abundance alterations observed in specific lipid classes during MSC senescence.

Table S1. All lipid annotations, *m/z*, and retention times for the first cohort.

Table S2. Annotations and *m/z* of all TG in the second MSC cohort**.**

Figure S3. Volcano plots of both donors distinguishing <10% senescence from >10% senescence.

Table S3. Key features distinguishing >20% senescent samples from <10% senescent samples in Donor 182.

Table S4. Key features to distinguish 10-20% senescent samples from <10% senescent samples in Donor 310.

Table S5. Table of the significant features in 10-20% senescence of both Donors.

Figure S4. Average fold change of each lipid class at every culture day, compared to the abundance at day 0, in both donors.

S0. Additional methods

Mass spectrometry quality assurance and annotations

All study cohort cell samples were randomized prior to LC-MS analysis. Blank samples of IPA and ammonium acetate were added to the run to reduce the effect of carryover throughout the analysis and to remove background features from sample spectra. All samples were spiked with Splash Lipidomix mix (Avanti). From the lipids in the Lipidomix mix, PC(15:0/18:1)(d7), was chosen for drift correction of all data in Compound Discoverer. The final concentration of PC(15:0/18:1)(d7) was 3 μg/mL. Quality control (QC) samples were used to account for instrument drift throughout the run, with QC injections performed after 8 sample injections. Drift corrections were applied correctly as the QC samples exhibited tight clustering in a PCA plot (data not shown). Additional multivariate analysis was performed using all unique features to identify similarities and differences to the metabolome of MSC at different culture times and between donors. Clustering between donors and MSC culture day can be seen in Figure S1, where there is separation between donors from all time points, however no distinct separation between MSC of varying culture days can be seen, showcasing the heterogeneity between donors.

Iterative data dependent acquisition (DDA) was also performed on QC material to help annotate unknown features. Confirmation of corrected instrumental drift MS/MS was performed on the most abundant features in an initial full scan MS to provide fragmentation data for features found in cell samples. All assigned annotations in this study were confirmed with accurate mass matching (<5 ppm) and MS/MS fragmentation data matching an in-house database created by the Georgia Tech Mass Spectrometry Core. Full lists of annotations from the first study cohort can be found in Table S1, and triglyceride annotations from the second study cohort are found in Table S2.

Lipidome comparisons between conditions

Following annotation of features within the run, an effort was made to identify patterns within the lipidome during MSC senescence. Polyunsaturated fatty acids are prone to lipid peroxidation which may lead to formation of oxidized lipids, compounds that have known roles in apoptosis and inflammation[1]. Oxidized lipid levels generally increase with age, leading to the creation of fold change plots for each lipid class by chain length and fatty acid unsaturation to search for a correlation between these characteristics and cellular age. Fold change was calculated from abundances of each lipid in samples of 10-20% senescence (from positive β-gal staining) to 0-10% senescent samples. As seen in Figure S2 the only notable pattern is a distinction between most lipid abundances and MSC donors, where Donor 182 exhibits a general decrease in lipid abundances from 0-10% senescence to 10-20% senescence, but the opposite trend appears in Donor 310 samples.

To further tease apart donor heterogeneity, volcano plots were created to observe the significant metabolites between higher senescent samples and lower senescent samples, as seen in Figure S3. Most lipids saw an increase in abundance as senescence progressed. Tables of significant lipids for both volcano plots are shown in Table S3 and Table S4. More specific shifts in lipid abundances

throughout MSC culture were observed in Figure S4, where trends are observed for each lipid class at every culture day. The average abundance of all lipids in each class were plotted, with culture day on the x-axis and fold change of the average class abundance at that culture day versus the average class abundance at day 0 was plotted on the y-axis. Similar trends are seen in samples from both donors, with a notable valley present in day 6 samples. Most lipid classes seemed to reach a minimum at day 6, when compared to day 0 data, before an abundance increase is observed for all lipid classes until the end of the study, day 12. Nearly all lipid classes, in both donors, saw a positive fold change at day 12 with the exception of ceramides in Donor RB310 exhibiting a slightly negative fold change.

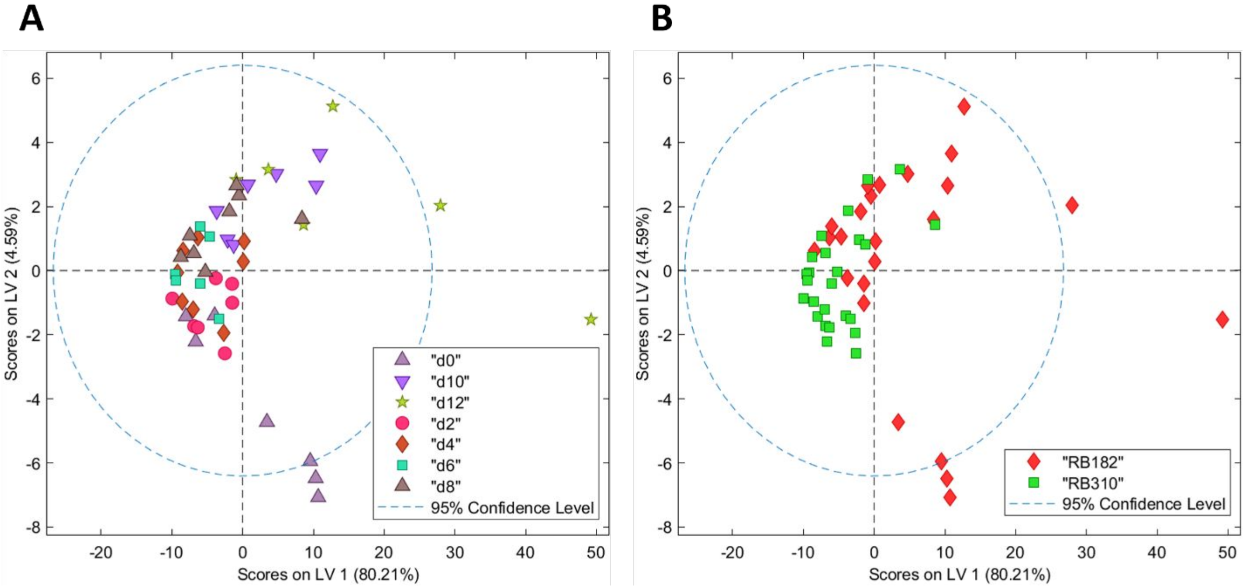

Figure S1. MSC undergo metabolism changes during standard culture. (A) PLSR scores plot using all observed LC-MS features colored by culture day, both donor lines; **(B)** PLSR scores plot, LV1 & LV2 colored by donor, RB182 (red diamond), RB310 (green square).

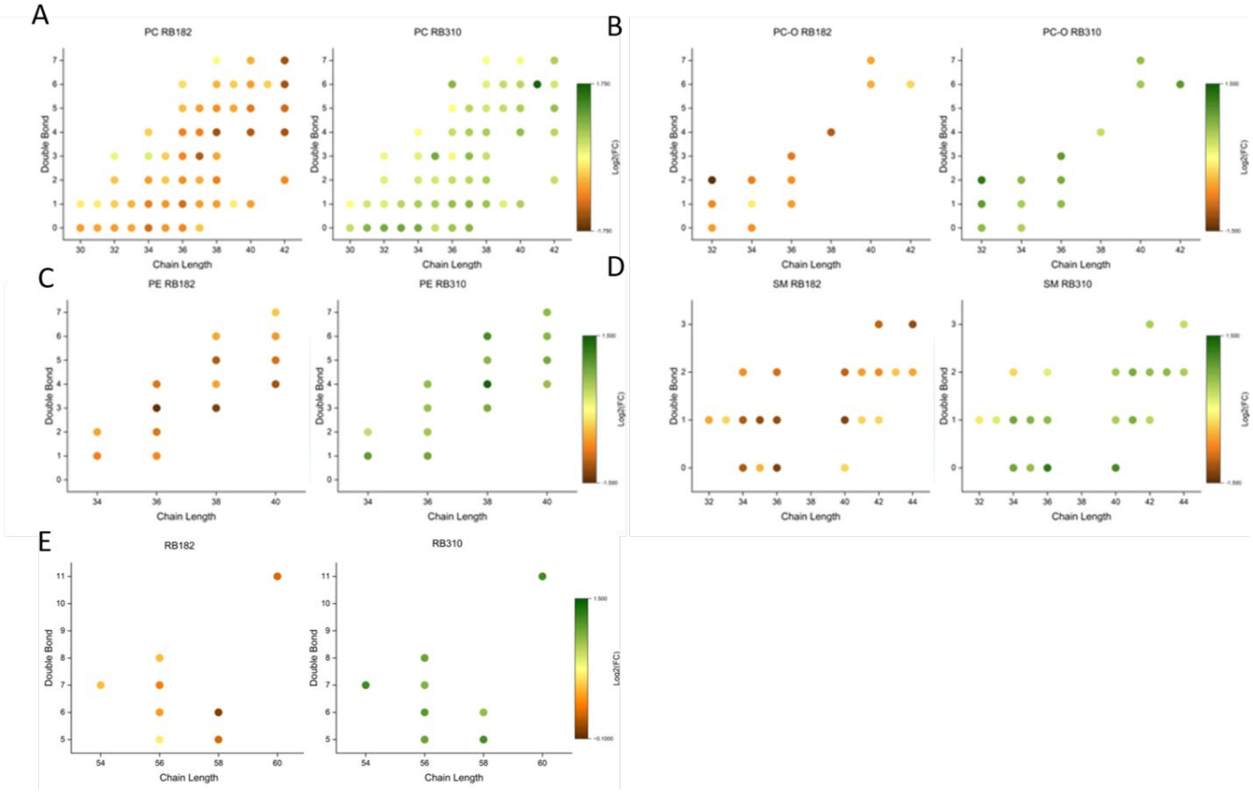

Figure. S2. Abundance alterations observed in specific lipid classes during MSC senescence. Calculated fold changes between 10-20% and <10% senescence MSC for lipids containing specific fatty acids with specific chain lengths and unsaturations for **(A)** PC, **(B)** PC-O, **(C)** PE, **(D)** SM, and **(E)** TG, for each donor. Color scale, Log_2(Fold change of (10-20% vs. <10%)), green indicates higher in 10-20% samples.

Table S1. All lipid annotations, *m/z*, and retention times for the first cohort. Annotations were confirmed using accurate mass and MS^2 spectral matching.

Name	*m/z*	RT [min]
PC(34:1)	760.586	5.694
PC(36:1)	788.617	6.637
PC(36:2)	786.601	5.725
PC(34:2)	758.570	4.999
PC(32:0)	734.570	5.660
PC(32:1)	732.554	4.826
PE(36:1)	746.571	6.489
PC(32:1)	732.555	4.997
SM(34:1)	703.575	4.621
PC(38:5)	808.585	4.829
PC(38:4)	810.601	5.758
SM(42:2)	813.685	7.018
PC(36:3)	784.585	5.027
PC(34:0)	762.601	6.626
PC(36:4)	782.570	4.797
PE(38:4)	768.554	5.666
PC(33:1)	746.571	5.235
PC(30:0)	706.538	4.725
PE(36:2)	744.555	5.631
PC(38:5)	808.585	5.001
PE(34:1)	718.539	5.584
PC(35:1)	774.601	6.253
PC(35:2)	772.585	5.281
PE(O-36:5)	724.528	5.164
PE(O-38:7)	748.528	4.956
PE(38:5)	766.538	4.772
PE(O-40:8)	774.543	4.991
PE(O-38:5)	752.560	6.138
PC(40:5)	836.616	5.805
PE(40:4)	796.585	6.294
PC(38:3)	812.617	6.208
SM(40:1)	787.669	7.106
PE(40:5)	794.569	5.702
PE(40:6)	792.554	5.415
PC(38:2)	814.632	6.610
PE(O-40:5)	780.591	6.620
PE(O-40:6)	778.575	6.174
PE(O-40:7)	776.559	5.877
Ganglioside GM3 (42:1)	1265.846	6.706
PC(O-34:1)	746.605	6.460
PC(40:6)	834.601	5.516
SM(35:1)	717.591	5.052
PC(38:6)	806.570	4.577
SM(42:1)	815.701	7.642
PE(38:5)	766.538	5.059
PE(38:6)	764.523	4.519
PC(38:4)	810.601	5.238
SM(34:1)	703.575	4.999
SM(42:3)	811.669	6.594
PC(40:6)	834.601	4.953
Hex3Cer(42:2)	1134.788	6.596
PE(O-34:2)	702.544	6.094
PC(38:4)	810.601	5.449
SM(34:0)	705.591	4.997
PC(34:2)	780.552	4.976
PS(40:5)	838.559	4.874
PE(40:7)	790.539	4.557
PS(40:6)	836.544	4.634
PC(31:0)	720.555	5.170
Cer(34:1)	538.520	5.332
PE(O-38:5)	752.560	5.820
Hex3Cer(42:1)	1136.803	6.935
PC(40:4)	838.632	6.466
PS(40:5)	838.559	4.996
PI(38:3)	889.581	5.022
HexCer(42:2)	810.682	6.811
SM(41:2)	799.669	6.784
PE(O-36:2)	730.575	6.743
PC(38:3)	812.617	6.474
PE(O-40:7)	776.559	5.240
PC(40:7)	832.586	4.603
DG(38:4)	627.535	4.693
PE(38:3)	770.570	6.053
HexCer(42:1)	812.698	7.130

Hex4Cer(42:1)	1339.883	6.855
PC(33:0)	748.586	6.181
PC(30:0)	706.539	4.999
PE(40:6)	792.554	4.806
PS(40:4)	840.575	5.444
PC(37:5)	794.606	5.493
SM(36:1)	731.607	5.587
PC(38:3)	812.615	5.947
PC(37:2)	800.617	6.220
PE(38:4)	768.554	5.322
DG(38:4)	662.572	6.746
Ganglioside GM3 (42:2)	1263.831	6.015
PC(37:3)	798.601	6.743
PC(34:3)	756.554	4.581
SM(40:2)	785.654	6.508
Cer(42:1)	650.645	7.462
Hex3Cer(40:1)	1108.772	6.655
PI(38:5)	885.549	3.968
HexCer(40:1)	784.667	6.825
PC(38:6)	806.570	4.314
PS(38:3)	814.560	5.194
PS(36:2)	788.544	5.000
PC(36:5)	780.554	4.282
SM(40:2)	785.655	6.681
PC(32:2)	730.539	4.554
PE(O-36:3)	728.560	6.094
PE(36:4)	740.523	4.733
PE(36:2)	744.555	5.828
PC(37:1)	802.632	6.908
PC(32:2)	730.539	4.158
PC(O-38:4)	796.622	6.497
PC(O-32:0)	720.591	6.476
PC(32:1)	732.554	6.078
PC(37:4)	796.586	5.268
PE(36:3)	742.539	5.040
PC(O-34:2)	744.591	6.317
PC(38:1)	816.648	7.152
PC(37:2)	800.617	6.880
Cer(42:3)	646.614	6.843
PC(33:0)	748.586	5.956
PC(37:4)	796.586	5.040

PC(31:0)	720.555	5.013
Hex3Cer(42:2)	1134.788	6.720
PE(34:2)	716.523	4.785
Cer(40:1)	622.614	7.139
LPC(18:0)	524.371	2.499
PI(40:5)	930.607	4.722
PC(O-34:0)	748.622	7.110
PE(O-40:5)	780.591	6.754
PC(36:2)	808.583	5.738
Ganglioside GM3(40:1)	1237.815	6.142
PS(38:4)	812.544	4.994
PC(O-36:2)	772.622	6.484
Hex3Cer(34:1)	1024.678	4.162
PC(30:1)	704.523	4.068
PC(34:1)	760.586	6.722
PC(34:3)	756.554	4.293
PC(37:3)	798.601	5.646
PC(36:5)	780.554	4.089
PI(38:3)	889.581	5.308
PC(39:5)	822.601	5.290
PI(40:5)	913.580	4.719
SM(41:1)	801.685	7.386
PC(35:1)	774.601	6.883
PE(O-40:6)	778.575	6.425
PC(35:3)	770.570	5.020
PE(O-37:5)	738.544	5.648
PC(31:1)	718.539	4.456
SM(d34:2)	701.560	3.959
PC(O-40:7)	818.606	5.195
PC(35:3)	770.570	4.717
SM(33:1)	689.560	4.231
PE(O-34:1)	704.559	6.735
PC(37:1)	802.632	7.189
PC(39:1)	830.664	7.511
SM(32:1)	675.544	3.864
TG(56:6)	924.802	8.670
Hex3Cer(41:1)	1122.788	6.812
LPC(18:1)	522.356	2.214
PC(O-32:1)	718.575	6.280
PC(42:5)	864.648	6.612
PC(40:2)	842.663	7.129

PS(40:6)	836.544	4.990
PC(35:2)	772.585	6.474
PC(42:4)	866.664	6.841
PS(36:2)	788.544	4.795
PC(O-42:6)	848.653	6.951
PI(38:5)	902.576	3.972
PS(36:1)	790.537	5.669
PC(O-40:6)	820.622	6.313
PE(O-40:4)	782.606	6.876
PC(O-40:6)	820.622	5.524
PC(35:0)	776.617	6.784
PC(42:7)	860.617	5.068
PC(36:0)	790.632	7.191
Cer(36:1)	548.540	6.307
PC(42:6)	862.632	5.741
PC(O-36:1)	774.638	7.096
Ganglioside GM3(41:1)	1251.831	6.512
TG(56:7)	922.786	8.504
PC(37:5)	794.606	6.306
SM(43:2)	827.700	7.257
Cer(41:1)	636.629	7.301
PC(O-32:2)	716.559	5.441
cholesterol	369.352	5.017
PC(34:2)	758.570	6.067
PI(36:2)	863.565	4.804
PC(34:4)	754.539	4.017
PE(38:3)	770.570	6.332
PC(38:7)	804.554	3.929
PC(O-35:2)	758.606	7.042
PC(40:5)	836.616	5.473
HexCer(34:1)	700.573	4.591
SM(36:2)	729.591	4.761
PC(O-36:3)	770.606	6.303
PC(39:5)	822.601	6.491
Cer(34:2)	520.509	5.337
SM(35:0)	719.570	4.260
TG(56:5)	926.818	9.093

PI(38:3)	906.607	5.030
PC(34:4)	754.538	5.179
Ganglioside GM3(44:2)	1291.862	6.648
SM(44:3)	839.700	7.079
PC(O-38:4)	796.622	6.183
SM(44:2)	841.716	7.542
PS(38:5)	810.528	4.086
TG(58:6)	952.833	9.104
PC(28:0)	678.507	3.951
PC(39:6)	820.585	4.599
Ganglioside GM3(43:1)	1279.862	6.824
PC(42:7)	860.617	5.459
PS(40:4)	840.623	4.792
SM(40:1)	809.654	5.959
PC(36:6)	778.538	4.738
TG(58:5)	954.849	9.386
Hex3Cer(44:2)	1162.819	6.889
PC(38:1)	816.702	7.187
PC(42:2)	870.695	7.654
HexCer(40:2-OH)	798.637	6.773
TG(56:7)	922.786	8.314
PI(34:2)	852.560	4.006
PC(40:1)	844.679	7.702
SM(40:0)	789.683	7.283
SM(36:0)	733.623	5.956
PE(O-40:4)	782.605	6.706
PC(32:3)	728.523	3.910
HexCer(44:2)	838.713	7.076
TG(54:7)	894.755	8.134
PC(37:0)	804.591	6.405
TG(56:8)	920.771	8.132
PE(O-36:5)	746.508	6.089
PC(39:6)	820.584	5.764
PC(39:6)	820.584	5.671
PC(41:6)	848.616	6.861
HexCer(43:1)	826.713	7.267

Table S2. Annotations and *m/z* of all TG in the second MSC cohort**.**

Annotation	*m/z*
TG(52:1)	878.817
TG(54:3)	902.817
TG(52:2)	876.801
TG(54:2)	904.833
TG(50:1)	850.786
TG(56:5)	926.817
TG(56:7)	922.786
TG(50:2)	848.770
TG(52:3)	874.786
TG(54:5)	898.786
TG(58:6)	952.832
TG(54:1)	906.848
TG(58:5)	954.848
TG(48:1)	822.755
TG(56:7)	922.786
TG(52:5)	870.755
TG(56:8)	920.770
TG(51:1)	864.801
TG(56:6)	924.802
TG(56:4)	928.833
TG(48:2)	820.739
TG(42:0)	740.677
TG(51:2)	862.786
TG(56:2)	932.864
TG(54:7)	894.755
TG(56:3)	930.848
TG(49:1)	836.770
TG(53:1)	892.833
TG(46:2)	792.708
TG(44:1)	766.692
TG(58:2)	960.895
TG(42:1)	738.661
TG(55:2)	918.848
TG(48:3)	818.724
TG(58:3)	958.879
TG(56:1)	934.880
TG(49:4)	830.721
TG(51:3)	860.770
TG(44:2)	764.677
TG(62:8)	1004.864
TG(64:13)	1022.817

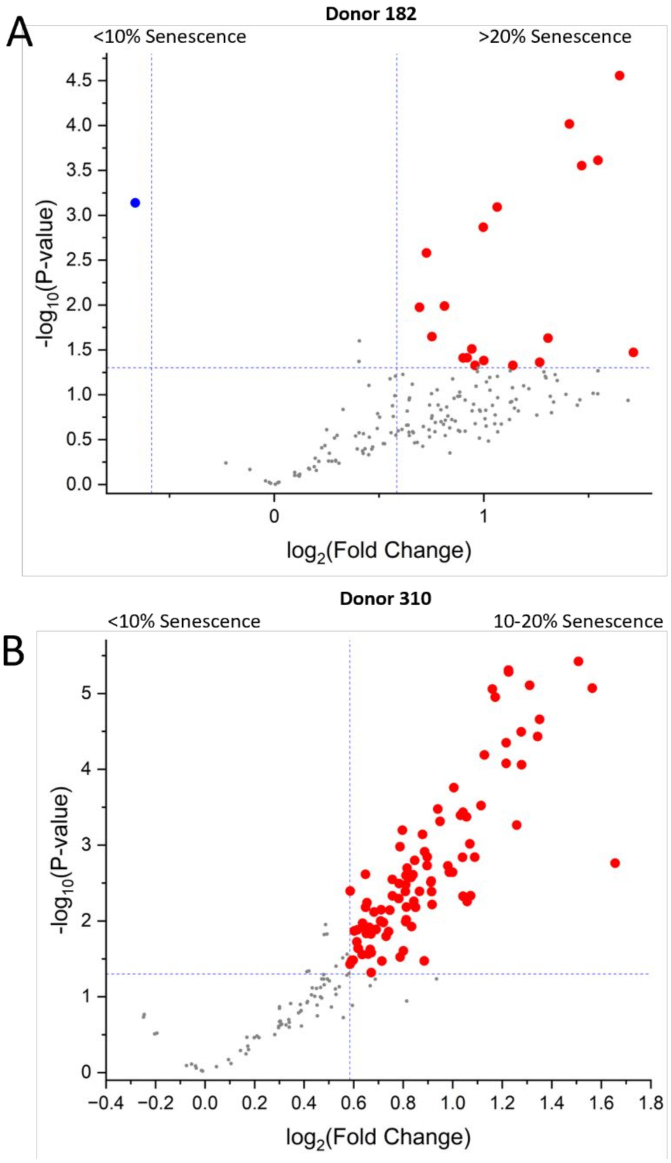

Figure S3. Volcano plots of **(A)** Donor 182 comparing >20% *vs*. <10% senescence and **(B)** Donor 310 comparing 10-20% *vs*. <10% senescence. Tables listing significant features can be seen in Tables S3 and S4.

Table S3. Key features distinguishing >20% senescent samples from <10% senescent samples in Donor 182. A volcano plot of this data is presented in **Figure S3A**.

**Donor 182**		
**Annotation**	**Fold Change**	**P-value**
TG(54:7)	3.135	2.782E-05
TG(60:11)	2.655	9.620E-05
TG(56:7)	2.918	2.445E-04
TG(56:7)	2.765	2.802E-04
PS(36:1)	0.632	7.273E-04
Hex3Cer(41:1)	2.091	8.115E-04
Hex3Cer(42:0)	1.997	1.360E-03
Hex3Cer(42:1)	1.655	2.629E-03
SM(41:1)	1.756	1.027E-02
Cer(41:1)	1.617	1.061E-02
Cer(40:1)	1.685	2.254E-02
HexCer(42:2)	2.473	2.338E-02
PC(39:1)	1.325	2.512E-02
SM(43:2)	1.923	3.093E-02
PC(O-42:6)	3.280	3.376E-02
PC(37:0)	1.893	3.869E-02
PE(O-36:2)	1.868	3.882E-02
Hex3Cer(42:2)	2.000	4.156E-02
SM(42:1)	1.323	4.245E-02
PE(O-40:8)	2.407	4.348E-02
PE(O-36:3)	1.943	4.706E-02
PE(O-40:7)	2.202	4.707E-02

Table S4. Key features to distinguish 10-20% senescent samples from <10% senescent samples in Donor 310. A volcano plot of this data is presented in **Figure S3B**.

Donor 310		
**Annotation**	**Fold Change**	**P-value**
Hex3Cer(42:2)	2.845	3.779E-06
PI(36:2)	2.338	4.922E-06
SM(36:0)	2.339	5.215E-06
Hex3Cer(42:0)	2.481	7.811E-06
Hex3Cer(41:1)	2.956	8.512E-06
PE(O-38:2)	2.235	8.753E-06
Hex3Cer(44:2)	2.253	1.118E-05
Hex3Cer(34:1)	2.551	2.199E-05
TG(60:11)	2.422	3.207E-05
Hex3Cer(40:1)	2.536	3.695E-05
Hex3Cer(42:1)	2.323	4.469E-05
PE(O-36:2)	2.186	6.470E-05
PE(O-34:2)	2.323	8.346E-05
TG(54:7)	2.425	8.717E-05
PI(40:5)	2.007	1.749E-04
PE(O-37:5)	2.166	3.008E-04
PC(O-42:6)	1.918	3.348E-04
TG(56:6)	2.295	3.461E-04
PE(O-36:3)	2.059	3.686E-04
PE(O-34:1)	2.044	4.033E-04
SM(40:0)	2.079	4.241E-04
PC(33:0)	1.930	4.868E-04
PC(O-32:2)	2.393	5.443E-04
PC(37:0)	1.737	6.349E-04
TG(58:5)	2.391	7.171E-04
PC(O-36:3)	1.838	7.228E-04
TG(56:5)	2.234	7.629E-04
TG(56:7)	2.098	9.637E-04
PI(34:2)	1.726	1.051E-03
TG(56:8)	2.216	1.066E-03
PE(O-38:5)	1.849	1.220E-03
TG(58:6)	2.050	1.370E-03
PC(O-32:1)	1.863	1.430E-03
PE(O-36:5)	2.127	1.442E-03
PE(38:6)	2.056	1.448E-03
PE(O-40:4)	1.798	1.593E-03
PC(41:6)	3.152	1.731E-03
PE(34:1)	1.862	1.866E-03
TG(56:7)	1.973	1.877E-03
HexCer(40:2-OH)	1.761	2.012E-03
PE(O-40:8)	1.982	2.276E-03
PI(40:5)	1.999	2.282E-03
SM(43:2)	1.567	2.431E-03
PE(40:6)	1.789	2.460E-03
PE(36:1)	1.755	2.512E-03
PE(O-40:7)	1.781	2.654E-03
SM(41:1)	1.689	2.834E-03
PC(34:0)	1.884	2.980E-03
PC(32:0)	1.882	3.079E-03
SM(41:2)	1.722	3.210E-03
SM(40:2)	1.753	3.324E-03
SM(35:0)	1.501	4.023E-03
PE(O-38:5)	1.823	4.071E-03
PE(40:5)	1.752	4.095E-03
HexCer(42:2)	1.886	4.112E-03
Cer(34:1)	2.103	4.653E-03
PC(O-36:2)	1.690	4.664E-03
PI(38:3)	2.059	4.728E-03
PC(37:1)	1.720	5.067E-03
Cer(42:3)	1.794	5.506E-03
PI(38:3)	2.082	5.526E-03
PC(38:1)	1.573	5.750E-03
PC(35:3)	1.888	6.074E-03
PC(31:0)	1.757	6.570E-03
PE(O-38:7)	1.802	6.596E-03
PC(O-36:1)	1.567	6.610E-03
PE(O-40:6)	1.637	7.133E-03
PC(37:3)	1.678	7.199E-03
PC(42:5)	1.605	7.624E-03
PE(36:2)	1.756	9.645E-03
SM(40:1)	1.635	1.003E-02
SM(34:0)	1.754	1.026E-02

PC(40:4)	1.647	1.047E-02
PC(O-32:0)	1.554	1.071E-02
PS(38:5)	1.783	1.186E-02
PE(O-40:7)	1.584	1.220E-02
Ganglioside GM3(41:1)	1.571	1.283E-02
PC(36:1)	1.614	1.296E-02
PC(O-40:7)	1.532	1.308E-02
SM(42:2)	1.518	1.355E-02
DG(38:4)	1.671	1.378E-02
PE(40:7)	1.574	1.379E-02
SM(36:1)	1.571	1.467E-02
PC(O-34:2)	1.591	1.489E-02
PS(38:3)	1.660	1.594E-02
Ganglioside GM3(43:1)	1.530	1.889E-02
PE(36:4)	1.535	2.300E-02

PE(38:5)	1.536	2.307E-02
PE(38:5)	1.588	2.376E-02
PC(36:6)	1.742	2.476E-02
PE(38:3)	1.592	2.622E-02
PS(40:6)	1.579	2.739E-02
PC(36:0)	1.553	2.771E-02
PS(38:4)	1.727	2.999E-02
PE(40:4)	1.514	3.286E-02
PC(40:5)	1.508	3.313E-02
Cer(34:2)	1.847	3.363E-02
SM(35:1)	1.641	3.374E-02
PC(34:1)	1.501	3.732E-02
Cer(42:1)	1.592	4.806E-02

Table S5. Table of the significant features in 10-20% senescence of both Donors from **Figure 4B**.

Annotation	Fold Change	P-Value
TG(54:7)	2.006	3.368E-05
Hex3Cer(42:0)	1.805	4.567E-05
TG(56:8)	1.926	1.712E-04
TG(56:6)	1.866	2.020E-04
TG(56:5)	2.070	2.772E-04
Hex3Cer(41:1)	1.825	3.690E-04
TG(60:11)	1.822	7.937E-04
TG(56:7)	1.687	8.692E-04
TG(56:7)	1.708	8.827E-04
Hex3Cer(42:1)	1.585	1.645E-03
TG(58:5)	1.798	3.091E-03
TG(58:6)	1.548	8.813E-03

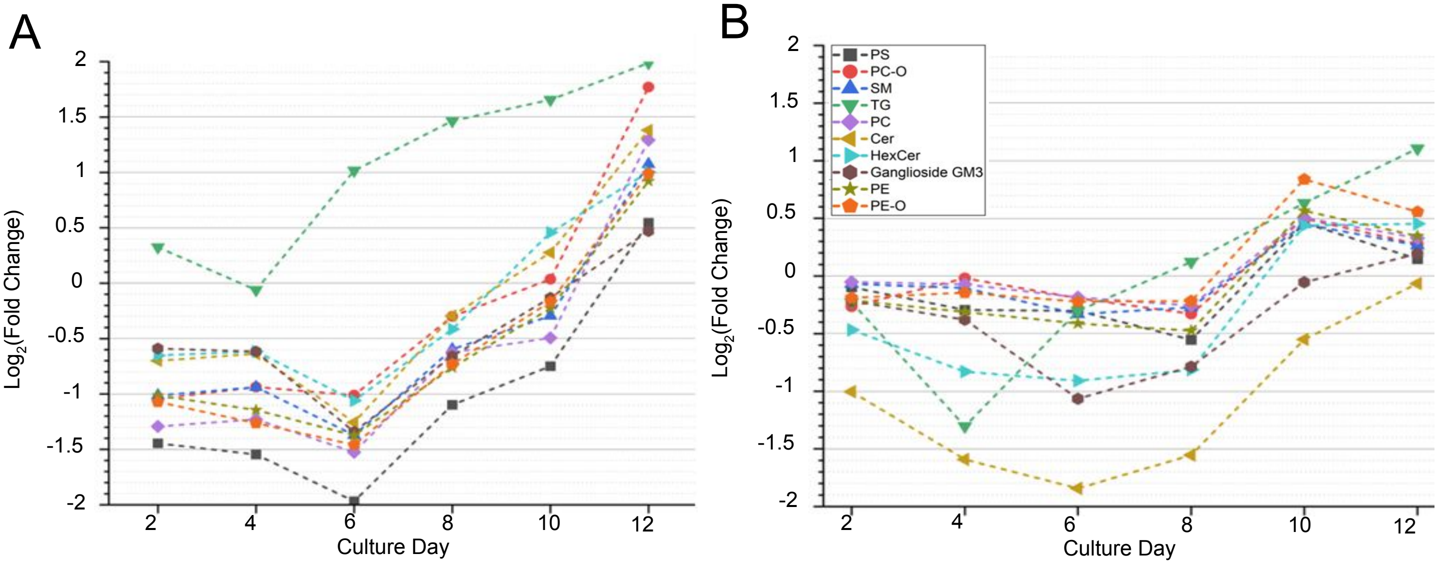

C

Lipid Class	Donor 182		Donor 310	
	Log2FC(D12/D2)	Log2FC(D6/D2)	Log2FC(D12/D2)	Log2FC(D6/D2)
TG	1.68	0.89	1.32	0.04
PC	2.58	-0.23	0.38	-0.13
PC-O	2.82	0.03	0.54	0.07
Ceramide	2.08	-0.56	0.94	-0.84
PE	1.93	-0.36	0.56	-0.20
PE-O	2.06	-0.39	0.75	-0.03
HexCer	1.58	-0.52	0.23	-0.84
PS	1.99	-0.52	0.24	-0.21
SM	2.09	-0.36	0.33	-0.26
Ganglioside	1.06	-0.75	0.41	-0.85
Hex3Cer	1.70	-0.34	1.48	-0.06

Figure S4. Average fold change of each lipid class at every culture day, compared to the abundance at day 0, in **(A)** Donor RB182 and **(B)** Donor RB310. A table of $Log_2(FC)$ values for each lipid class can be seen in **(C).**

References

[1] Hamsanathan, S., & Gurkar, A. U. Lipids as Regulators of Cellular Senescence. Frontiers in physiology, *Front Physiol* **2022,** 13, 796850.
